# Tectorigenin Ameliorates Glucocorticoid‐Induced Osteoporosis by Inhibiting the NF‐κB Signal Pathway and Modulating Treg‐Th17 Cell Balance

**DOI:** 10.1111/jcmm.70705

**Published:** 2025-07-06

**Authors:** Peng Peng, Puiian Wong, Zheng Lv, Jiaqing Tian, Wei He, Qiushi Wei, Hui Mo, Mincong He

**Affiliations:** ^1^ Department of Orthopedics Guangdong Provincial Second Hospital of Traditional Chinese Medicine Guangzhou China; ^2^ State Key Laboratory of Traditional Chinese Medicine Syndrome Guangzhou China; ^3^ Guangdong Provincial Engineering Technology Research Institute of Traditional Chinese Medicine Guangzhou China; ^4^ Guangdong Provincial Key Laboratory of Research and Development in Traditional Chinese Medicine Guangzhou China; ^5^ Faculty of Chinese Medicine Macau University of Science and Technology Macau China; ^6^ The First People's Hospital of Zhaoqing Zhaoqing China; ^7^ Guangdong Research Institute for Orthopedics and Traumatology of Chinese Medicine Guangzhou Guangdong China; ^8^ State Key Laboratory of Traditional Chinese Medicine Syndrome/Orthopaedic The Third Affiliated Hospital of Guangzhou University of Chinese Medicine Guangzhou Guangdong China; ^9^ Department of Orthopaedics, the Third Affiliated Hospital Guangzhou University of Chinese Medicine Guangzhou China; ^10^ Macao Health Bureau WHO Traditional Medicine Cooperation Center Macau China

**Keywords:** osteoclast, osteoporosis, RANKL, tectorigenin, Th17/Treg

## Abstract

Glucocorticoid‐induced osteoporosis is a common clinical orthopaedic disease, primarily due to excessive use of hormones. Tectorigenin has shown a certain efficacy in treating osteoporosis, but its underlying mechanisms are not yet fully understood. This study aimed to evaluate tectorigenin (TEC)'s potential in treating glucocorticoid‐induced osteoporosis (GIOP) by restoring the osteoclast–osteoblast balance. By employing molecular docking to study TEC–RANKL interaction and RNA sequencing to map TEC's anti‐osteoporotic pathways, we conducted in vitro tests on TEC's effects on osteoclast differentiation, bone resorption, ROS production, and NF‐κB activation. We performed a luciferase reporter assay for Nrf2 and NF‐κB activities and evaluated TEC's in vivo efficacy using a GIOP mouse model with micro‐CT and histomorphometric analyses, measuring serum RANKL levels and Treg/Th17 cell ratios. Docking analysis confirmed TEC's specific binding to RANKL and RNA sequencing showed TEC modulated osteoclast pathways. In vitro, TEC suppressed osteoclastogenesis, bone resorption, osteoclast gene expression, and ROS activity by downregulating NFATc1 and modulating NF‐κB. The luciferase assay revealed TEC inhibited Nrf2 and influenced its interaction with NF‐κB. In vivo, TEC protected against GIOP by balancing Th17/Treg cells to inhibit osteoclast differentiation and maintain bone volume. In conclusion, tectorigenin (TEC) shows promise in reducing osteoclastogenesis and preventing GIOP, making it a potential drug candidate for osteoporosis management.

## 1 Introduction

Osteoporosis, a common disease characterised by the deterioration of bone microarchitecture and a reduction in bone mass, can be classified into primary and secondary types [1]. Glucocorticoid intake is one of the most common causes of secondary osteoporosis [2]. Glucocorticoids are widely used to effectively treat inflammatory and immune‐mediated disorders such as rheumatoid arthritis and asthma [3]. However, clinical research has shown that almost 50% of patients taking glucocorticoids for more than 6 months develop osteoporosis [4]. Moreover, for patients who have received glucocorticoid treatment for more than 5 years, the incidence rate of fractures is as high as 30% [5]. Previous research has shown that excess glucocorticoids can prolong the life span of osteoclasts and enhance osteoclastogenesis via upregulating RANKL in osteoblasts [6, 7]. In addition, Kaji et al. demonstrated that dexamethasone could stimulate osteoclast‐like cell formation in the stromal cell‐containing mouse bone cell cultures in a concentration‐dependent manner [8]. The hyperactivity of osteoclast differentiation and life span is the key to the pathogenesis of osteoporosis [9].

It has been found that the receptor activator of nuclear factor‐B ligand (RANKL), a member of the tumour necrosis factor (TNF) ligand family, can bind to its receptor RANK on the surface of osteoclasts and stimulate osteoclast maturation and differentiation [10]. The action of RANKL is blocked by osteoprotegerin (OPG), which prevents the binding of RANKL to the RANK receptor and subsequently inhibits bone resorption [11]. RANKL‐ and RANK‐deficient mice have been shown to exhibit severe osteopetrosis, accompanying a lack of osteoclast differentiation [12, 13]. In contrast, OPG‐deficient mice exhibit severe osteoporosis arising from enhanced adult‐stage osteoclastogenesis [14]. Accordingly, OPG and soluble RANK have been investigated as potential therapeutic targets, and an anti‐human RANKL antibody called denosumab has been employed in the clinical setting for the treatment of osteoporosis and cancer‐related bone disorders [15].

Tectorigenin (TEC), an O‐methylated isoflavone, is a natural extraction and main active ingredient of *Belamcanda chinensis* . Previous studies report that it has potential usage in antioxidant and anti‐inflammatory [16]. Lee et al. reported that TEC promoted the osteogenic differentiation of primary osteoblasts and periodontal ligament cells. Moreover, TEC upregulated the expression of the BMP2, BMP4, and Smad‐4 genes, and enhanced the expression of Runx2 and Osteri [17]. Although TEC is considered a promising therapy for postmenopausal osteoporosis in mice models, the effects of this compound on glucocorticoid‐induced osteoporosis (GIOP) have not been reported to date [18]. In this study, the effect of TEC on RANKL‐induced ROS level, NF‐κB, and NFATc1 in vitro and osteoclastogenesis was investigated. Furthermore, its anti‐ GIOP effect in the mice model was studied in vivo.

## 2 Materials and Methods

### 2.1 Protein‐Ligand Molecular Docking

The 2D structures of TEC and the crystal structure of the target RANKL‐RANK complex were obtained from the PubChem and PDB databases. A model with ligand binding smaller than 3 Å was selected for analysis. The crystal structure was then imported into the Pymol 1.7.2.1 Software (https://pymol.org/2/) for dehydration, hydrogenation, and ligands separation. The docking grid for RANKL was generated using AutoDockTools 1.5.6 [19, 20]. Subsequently, the docking process was performed with AutoDock software. Docking was completed by AutoDock software. The docking conformation with the lowest binding energy was chosen to evaluate the binding efficacy by examining the ligand and intermolecular interactions, such as hydrophobic interactions.

### 2.2 RNA Sequencing

RAW 264.7 cells that had been stimulated with RANKL for 2 days were cultured with TEC (200 μM) in comparison to a control group. Total RNA was extracted on the second day of the culture period by homogenising cells from the TEC group (*n* = 3) and control group (*n* = 3) in TRIzol reagent. Each sample utilised 1 μg of RNA as input material for RNA sample preparations. Illumina sequencing libraries were generated, and index‐coded samples were clustered using a BotCluster Generation System with PE Cluster Kit cBot‐HS (Illumina) for further analysis. The library preparations were sequenced using an Illumina HiSeq platform to generate paired‐end reads. The filtered reads were aligned to the *Mus musculus* genome (Mus_musculus. GRCm38) using HISAT2 (version 2.0.5). HTSeq (version 0.6.1) was utilised to quantify the read numbers mapped to each gene. Differential expression analysis between two groups was conducted with the DESeq2 R package (version 1.20.0). Expression of which |fold change (FC) | ≥ 1.5 and *p* < 0.05 were screened out as differential of expressed genes (DEGs).

### 2.3 Functional Enrichment Analysis and Protein–Protein Interaction (PPI) Network Construction

Functional enrichment analysis of DEGs including Gene Ontology (GO) and Kyoto Encyclopedia of Genes and Genomes (KEGG) enrichment analysis was performed using the DAVID database (https://david.ncifcrf.gov/). The *p* < 0.05 was considered significant. The DEGs were then uploaded into the online database STRING (https://string‐db.org/) to construct a protein–protein interaction (PPI) network. The interaction score threshold was set to a medium confidence level (> 0.4), and isolated nodes were removed. Subsequently, the network was imported into Cytoscape software (version: 3.9.1), and hub shared genes were filtered.

### 2.4 Preparation of Drugs and Reagents

The Standard of TEC (purity > 98% by HPLC) was obtained from WEIKEQI, CHINA. TEC was diluted in DMSO and stored at −20°C. We set up different TEC concentrations (100 μm, 200 μm) for further experiments.

### 2.5 Cell Viability Assay

The cytotoxicity of TEC was assessed using the CCK‐8 assay in accordance with the manufacturer's instructions. Briefly, RAW 264.7 cells were plated in 96‐well plates with a density of 3000 cells per well. Following the intervention of TEC at 24 or 48 h, cell viability was determined using the CCK‐8 method and absorbance was measured using a Multiscan Spectrum spectrophotometer 1 h after the addition of 10 μL of CCK8 reagent (Fdbio, CHINA) to each well.

### 2.6 Osteoclasts Differentiation

RAW264.7 cells were utilised to assess the ability of osteoclast differentiation by TEC. The differentiation of mature multinuclear osteoclasts was evaluated using TRAP staining, with 3000 cells seeded per well in a 96‐well plate and exposed to 50 ng/mL of RANKL. The induction medium was replenished every 2 days until multinuclear osteoclasts were observed. Following fixation with 4% PFA for 30 min and washing with PBS three times, the cells were incubated with TRAP staining solution at 37°C for 1 h. Images were captured in three randomly selected areas using a microscope, and quantitative analysis was performed using Image J.

### 2.7 qRT‐PCR

RAW264.7 cells were seeded in 12‐well plates at a density of 1 × 106 cells/well and treated with TEC at concentrations of 100 or 200 μmol/L in the presence or absence of RANKL (50 ng/mL) for 48 h. We used TRIzol reagent (Invitrogen, Carlsbad, CA, USA) to extract RNA from cells according to the protocol. Total RNA was used to synthesise cDNA by reverse transcription (cDNA Synthesis Kit, Takara Biotechnology, Otsu, Japan). Power SYBR Green PCR Master Mix (Takara Biotechnology) was used for real‐time PCR. The expression of TRAP, NFATc1, CTSK and RANK were analysed using primer sequences listed in Table 1. GAPDH served as the endogenous control. For real‐time PCR, a 10‐μL reaction mixture contained SYBR Green and each primer. The program included one cycle of denaturation at 95°C for 1 min and 40 cycles of denaturation at 95°C for 15 s, primer annealing and extension at 63°C for 25 s, followed by melt curve analysis. Data were analysed for fold difference using the formula: 2‐^ΔΔ^CT.

**TABLE 1:** The primers of each factor.

TRAP	F:5′‐CACTCCCACCCTGAGATTTGT‐3′
	R:5′‐CATCGTCTGCACGGTTCTG‐3′
NFATc1	F:5′‐GGAGAGTCCGAGAATCGAGAT‐3′
	R:5′‐TTGCAGCTAGGAAGTACGTCT‐3′
CTSK	F:5′‐CTCGGCGTTTAATTTGGGAGA‐3′
	R:5′‐TCGAGAGGGAGGTATTCTGAGT‐3′
RANK	F: 5′‐TCCTCGGGAACTGGCTATG‐3′
	R:5′‐GGTTGGGTCCCATTGGAGAC‐3′

### 2.8 Western Blot Analysis

Cells were cultured in 25 cm^2^ culture dishes and subjected to TEC intervention for varying durations (3 or 5 days, or 20 or 60 min). Following this, the cells were lysed in a solution containing RIPA, PMSF (1: 100), and phosphatase inhibitors (1: 100). The resulting suspension was incubated in EP tubes for 30 min and centrifuged at 12000 rpm for 20 min at 4°C. The supernatant was collected and its concentration determined using a Bradford assay kit (Solarbio, CHINA). Subsequently, the sample was prepared with 5× loading buffer. Following the preparation of running gels, the samples were electrophoresed in the upper gel at 80 V for 20 min, followed by electrophoresis in the lower gel at 100 V for 80 min. PVDF membranes were transferred at 55 V for 75 min. The PVDF membranes underwent blocking in QuickBlock Western buffer (Beyotime, CHINA) and were then subjected to incubation with primary antibodies (1: 1000) at 4°C overnight. The following day, secondary antibodies (1: 5000) were incubated, followed by washing in TBST buffer and development using ECL solution (Beyotime, CHINA).

### 2.9 GIOP Mice Model Establishment and Treatment

Purchased 24 C57BL/J female mice from Laboratory Animal Center, Guangzhou University of Traditional Chinese Medicine (GUANGZHOU, CHINA); approximately 8 weeks old. The mice were divided into four groups: control group (intramuscular injection of normal saline and intraperitoneal injection of PBS, *n* = 6), GIOP group (intramuscular injection of Dex and intraperitoneal injection of PBS, *n* = 6), GIOP+TEC (intramuscular injection of Dex and intraperitoneal injection of 10 mg/kg TEC, *n* = 6) group and GIOP+TEC (intramuscular injection of Dex and intraperitoneal injection of 20 mg/kg TEC, *n* = 6) group. The osteoporosis model was established by intramuscular injection of Dex (100 mg/kg/day) on alternate days for the first 4 weeks [21]. After 4 weeks, DEX was injected every other day to maintain the modelling effect, and low‐concentration and high‐concentration TEC were intraperitoneally injected; all mice were fed freely. The modelling and drug administration process spanned a duration of 6 weeks, after which the mice were euthanised and dissected. Limbs, particularly those of the knee joint, were preserved in neutral formalin for a period of 2 days before being transferred to 75% alcohol for subsequent experimentation. Animal experiments were performed in compliance with the International Guiding Principles for Biomedical Research Involving Animals and were approved by the Institutional Animal Care and Use Committee of the Guangzhou University of Chinese Medicine (20210202002).

### 2.10 Flow Cytometry Analysis

To analyse the percentage of Treg and Th17 cells in splenocytes, spleens were isolated from the individual mouse in each group and ground to obtain the cell suspension. The cell suspension was passed through a sterile filter to remove the debris, and red blood cell lysis buffer was used to lyse the red blood cells. After centrifugation, the cell suspensions were washed twice with PBS and stored in PBS containing 3% fetal bovine serum on ice. The Treg cells were marked with CD25+ CD4+ Foxp3 + antigens, stained with FITC‐conjugated anti‐CD4, APC‐conjugated anti‐CD25 and PE‐conjugated anti‐Foxp3. The Th17 cells were marked with CD4+ IL‐17 antigens, and the cell suspensions were first treated with ionomycin and phorbol myristate acetate (PMA)for 1 h in 5% CO2 37°C and then stained with FITC‐conjugated anti‐CD4 and PEcy7‐conjugated anti‐IL‐17. Finally, the stained cells were detected by using a flow cytometry assay.

### 2.11 Micro‐CT Scan and Analysis

Following the replacement of 75% alcohol, the limbs were encased in a cylindrical tube to secure their position. Subsequent scanning was conducted using the Skyscan 1176 micro‐CT scanner (Bruker micro‐CT, Kontich, Belgium) with the following parameters: voltage set at 60 kV; source current at 417 μA; Al 0.5 mm filter; pixel size of 9 μm; rotation step of 0.5°. A region of interest for trabecular bone analysis was defined as 0.009 mm below the growth plate on the distal tibia, with a height of 0.27 mm. The parameters of trabecular bone were analysed, including bone mineral density (BMD), bone volume/tissue volume (BV/TV), trabecular number (Tb. N), trabecular pattern factor (Tb. Pf), trabecular thickness (Tb. Th) and trabecular separation (Tb. Sp).

### 2.12 Bone Histomorphometry, Tartrate‐Resistant Acid Phosphatase(TRAP) Staining and Immunohistochemistry

Osseous tissue was collected and fixed in 4% formaldehyde for 48 h. Bone tissues were then decalcified, dehydrated and inserted into paraffin wax to cut 5‐μm‐thickness sections. They were stained by haematoxylin and eosin (H&E) (Solarbio, CHINA) and tartrate‐resistant acid phosphatase (TRAP) (Sigma, St Louis, MO, USA). In addition, the 5‐μm‐thickness sections were then immuno‐stained using the antibodies of rabbit anti‐RANKL (1:100; Proteintech Group Inc). The BIOQUANT OSTEO software (Bioquant Image Analysis Corporation, Nashville, TN, USA) was used in bone histomorphometric analysis.

### 2.13 Enzyme‐Linked Immunosorbent Assay (ELISA)

In accordance with the manufacturer's protocol, the levels of the RANKL in plasma were quantified via an Enzyme‐Linked Immunosorbent Assay (ELISA) kit (Abcam, catalogue number ab100749, Cambridge, UK). Plasma samples were dispensed into the designated assay wells and subsequently combined with both standard and blank wells. The microtiter plate was then incubated for a period of 1–2 h at a temperature of 37°C, followed by a thorough washing procedure using a dedicated washing buffer. Post‐washing, a dilution of biotinylated detection antibodies and enzyme conjugates was prepared, which was then subjected to pre‐warming and subsequent wash cycles. The chromogenic substrate, TMB, was added and allowed to incubate for precisely 5 min. The enzymatic reaction was terminated through the addition of a stop solution. Finally, the absorbance readings were obtained at a wavelength of 450 nm utilising a microplate reader.

### 2.14 Statistical Analysis

Statistical analysis was performed using SPSS software version 23.0, with results presented as mean ± standard deviation (mean ± SD). Data adhering to a normal distribution were compared using Student's *t*‐test or one‐way ANOVA, while non‐normally distributed data were assessed using the Mann–Whitney *U*‐test. *p* < 0.05 indicates statistical significance.

## 3 Results

### 3.1 Molecular Docking: Identification of Affinity of TEC‐RANKL Complex

**FIGURE 1:**
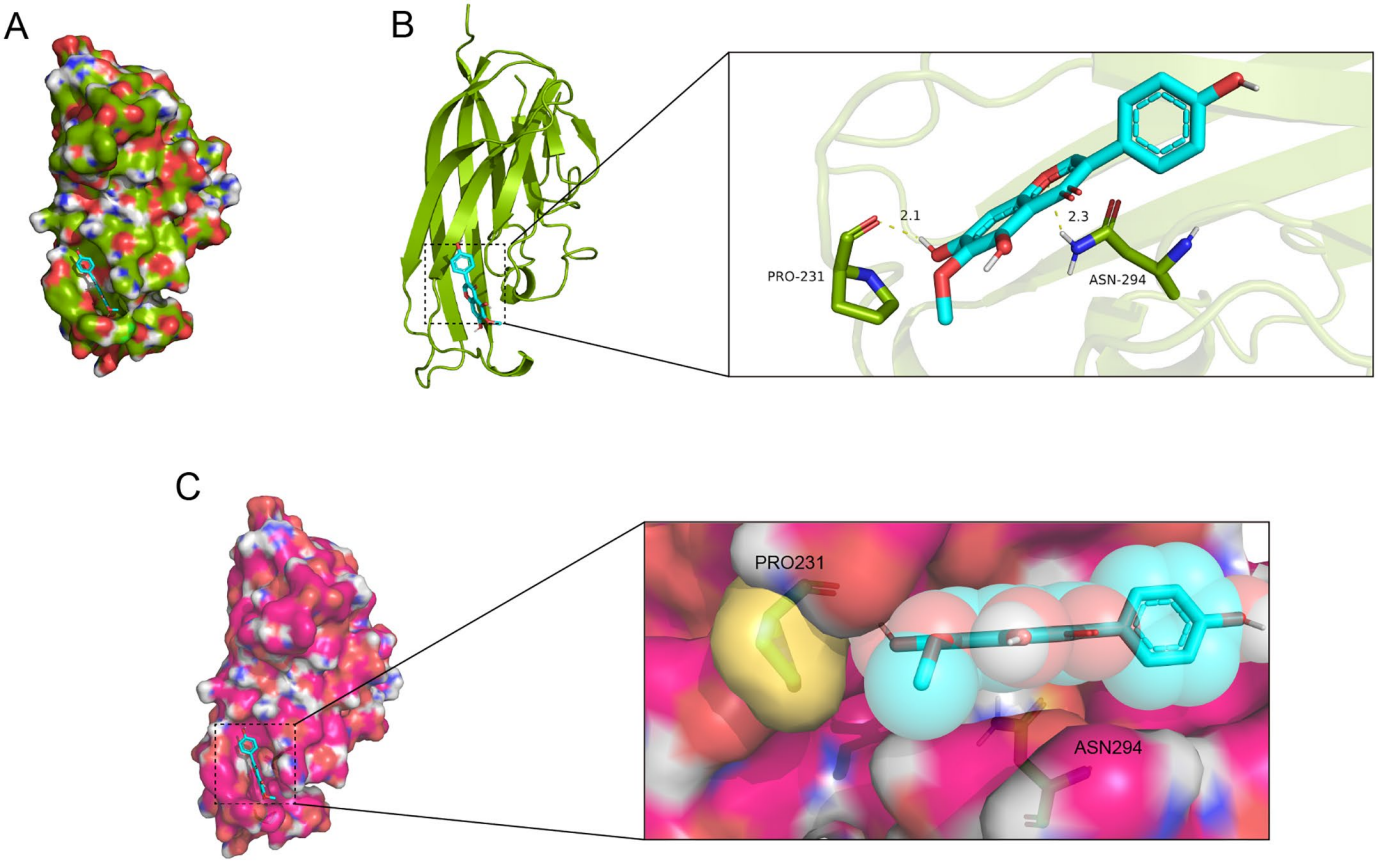
Construction of the RANKL crystal model. (A) A potential binding site for TEC within a hydrophobic domain, encompassing both hydrogen bond acceptors and donors, was computationally predicted. (B) The initiation point of the binding site for TEC was identified at residue PRO231, extending towards ASN294. (C) Computational docking simulations revealed a robust affinity between TEC and RANKL.

A crystal model of RANKL was created to serve as a foundational structure for TEC matching, as displayed in Figure 1A. Through computational analysis, a potential binding site for TEC within a hydrophobic region containing both hydrogen‐bond acceptor and donor functionalities was identified in Figure 1B. The critical region for TEC binding initiation was determined to span from residues PRO231 to ASN294. Computational docking simulations revealed a significant affinity between TEC and RANKL, with a calculated binding free energy of −6.5 kcal/mol. Overall, our findings suggest that the robust interaction between TEC and RANKL is primarily driven by their complex non‐covalent interactions.

### 3.2 Differential Expression Analysis in TEC‐Treated and Control Group

**FIGURE 2:**
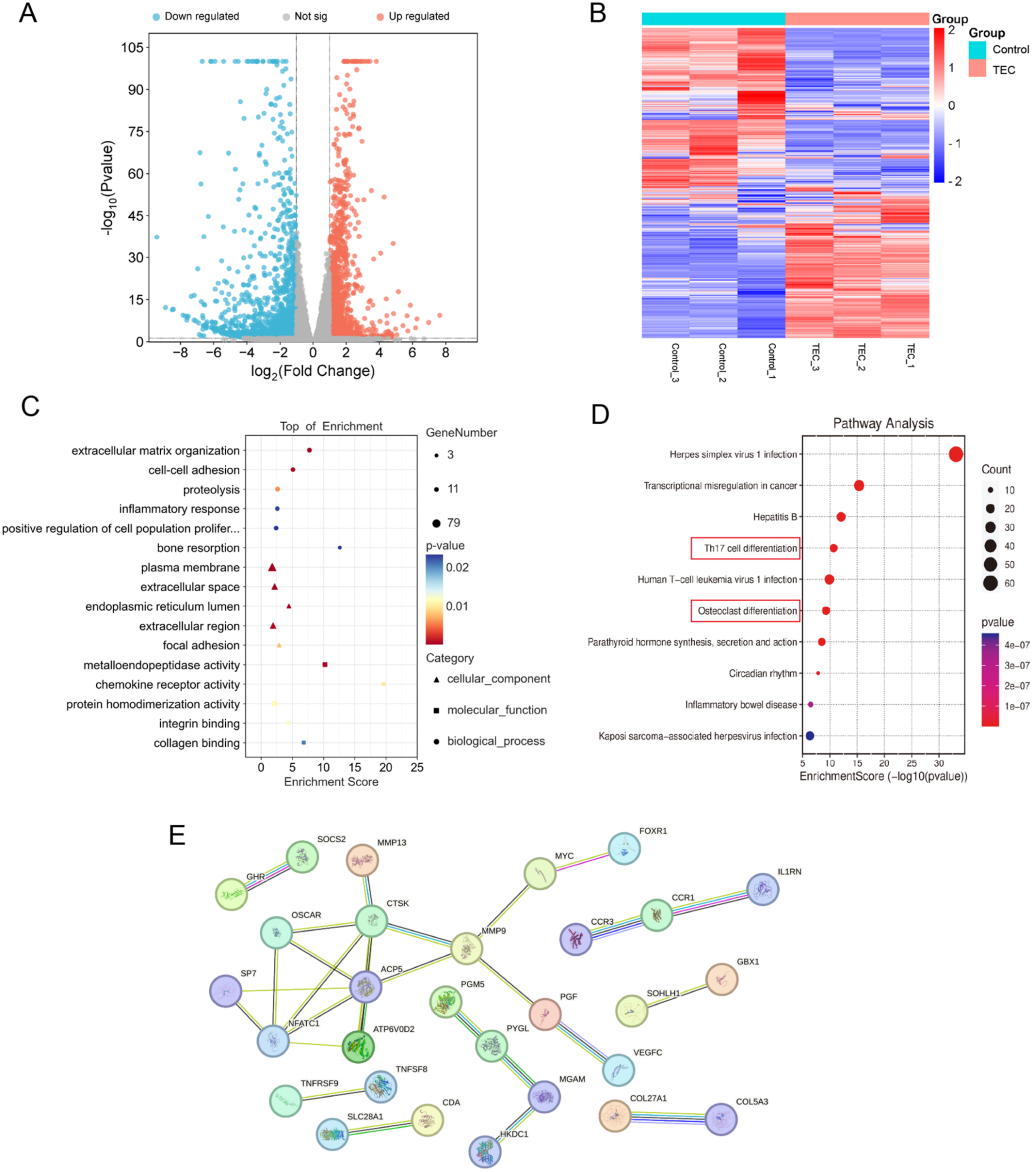
RNA‐Seq analysis revealed a total of 597 genes exhibiting upregulation and 1010 genes showing downregulation (A). The volcano and heatmap plots for RNA sequencing data were generated (B). To delve deeper into the functional implications of these gene expression changes, we conducted Gene Ontology (GO) enrichment analysis focusing on biological processes (BPs), molecular functions (MFs) and cellular components (CCs), providing a comprehensive understanding of the underlying biological mechanisms (C). Additionally, we performed Kyoto Encyclopedia of Genes and Genomes (KEGG) pathway analysis to pinpoint the top 10 signalling pathways impacted by these differentially expressed genes (D). Lastly, we constructed and graphically represented a protein–protein interaction (PPI) network centred around the co‐regulated downregulated genes, offering a visual framework for their interconnectedness (E).

RNA sequencing analysis identified 597 upregulated and 1010 downregulated DEGs in the TEC‐treated group compared to the control groups. The volcano and heatmap plots for RNA sequencing data were generated (Figure 2A,B). To further understand the impact of DEGs, GO enrichment analysis focusing on biological processes (BPs), cellular components (CCs) and molecular functions (MFs) were conducted (Figure 2C). The analysis indicated that DEGs primarily influenced BPs related to extracellular matrix organisation, cell–cell adhesion, proteolysis and inflammatory processes. The primary CCs affected were plasma membrane, extracellular space and endoplasmic reticulum lumen. The major MFs involved were metalloendopeptidase activity, chemokine receptor activity, protein homodimerisation activity and integrin binding. KEGG pathway analysis was conducted to identify the top 10 signalling pathways influenced by the DEGs (Figure 2D). Notably, the DEGs exhibited significant influence on the osteoclast differentiation pathway (red frame in Figure 2D). A PPI network was constructed to analyse coregulated downregulated genes, as depicted in Figure 2E. The presence of NFATc1 and ACP5, which exhibit numerous connections within the network, implies their potential as key regulatory molecules.

### 3.3 TEC Suppresses RANKL‐Induced Osteoclasts Formation

**FIGURE 3:**
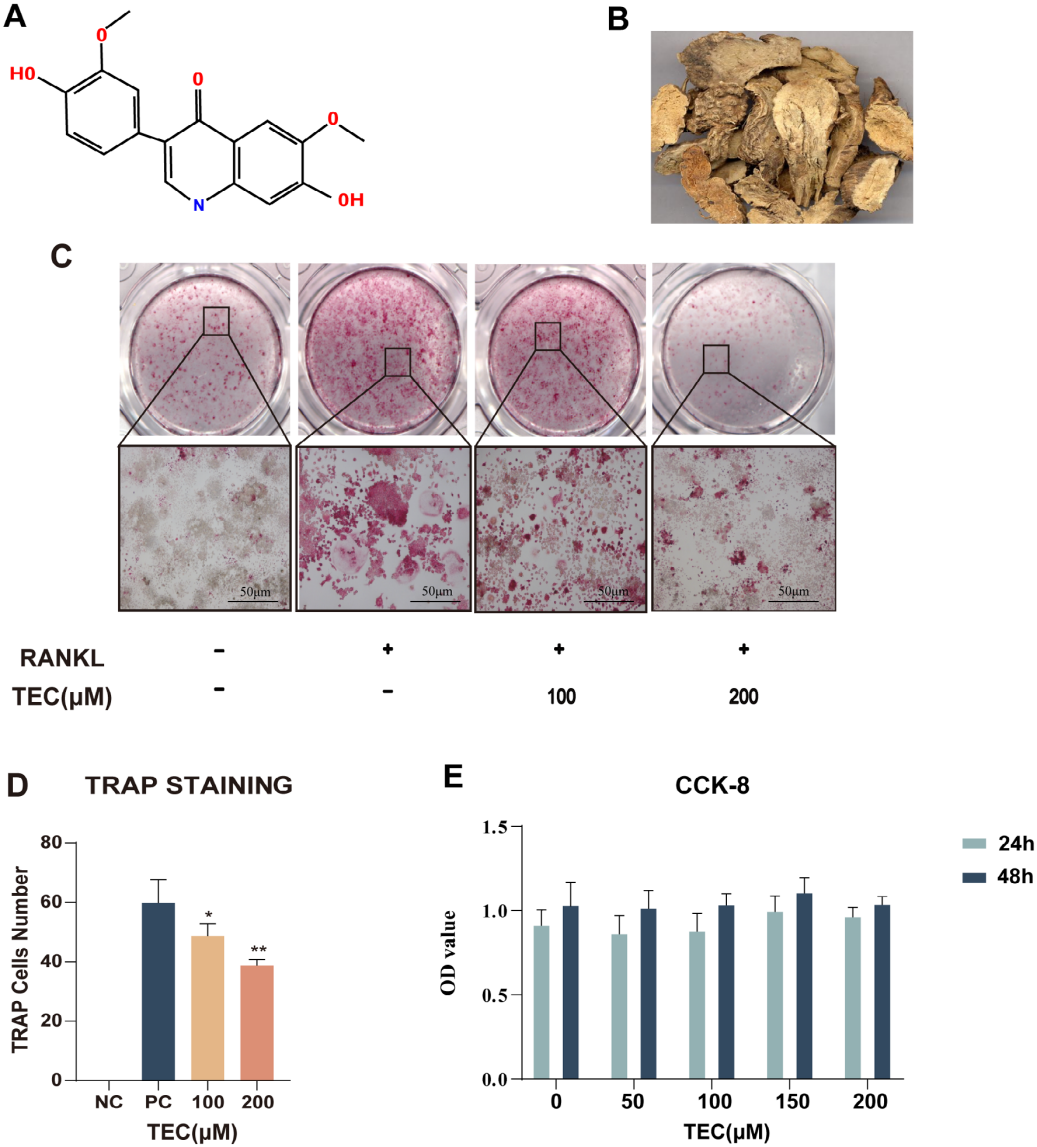
TEC reduces RANKL‐induced osteoclast differentiation in vitro without cytotoxicity. (A) The molecular formula of tectorigenin. (B) Graphic of tectorigenin source. (C) TRAP staining of osteoclast induced by RANKL. (D) Quantification analysis of TRAP‐postice staining in RAW 264.7 cells. (E) CCK‐8 assay for cytotoxicity of RAW 264.7 cells cultured on TEC at both 24 and 48 h. All bar charts are presented as the mean ± SD; *n* = 4; scale bar = 500 μm. *p < 0.05, **p < 0.001, relative to the non TEC treatment group.

The molecular formula was shown as follows (Figure 3A,B). The cell viability and cytotoxicity were measured by the CCK‐8 kit. We set up 24 h and 48 h to assess if different concentrations of TEC (50 μM, 100 μM, 150 μM, 200 μM) can affect the viability of RAW264.7 cells (Figure 3E). No statistically significant differences were observed in cell viability across the different concentrations tested, suggesting that TEC's differentiation ability is independent and forms the basis for further experimentation. Meanwhile, TEC concentrations (100 μM,200 μM) were selected from four different concentrations for the osteoclast formation assay (Figure 3C,D), with the criterion for TRAP staining being the presence of three or more multinucleated giant osteoclasts. Following 96 h of RANKL induction, our results indicated that TEC inhibits osteoclast formation in a dose‐dependent manner, as evidenced by a reduction in the number of mature osteoclasts compared to the positive control. Notably, the 200 μM TEC concentration exhibited the most significant inhibition of osteoclast formation (Figure 3).

### 3.4 TEC Inhibits the Expression of Inflammatory Factors and Downregulate Specific Osteoclast Differentiation Genes via NF‐kB Signalling

**FIGURE 4:**
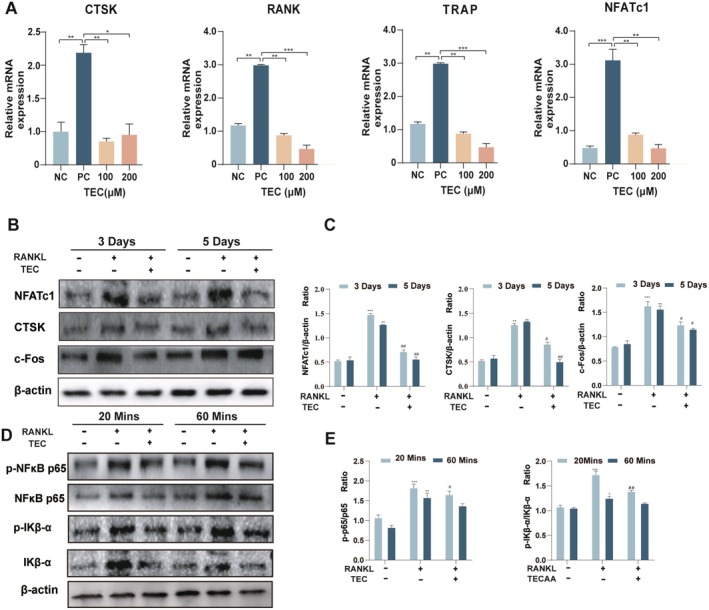
Tec inhibits the NF‐κB/IKB‐α pathway and suppresses the expression of NFATc1 and CTSK, which are further associated with specific osteoclastogenesis genes (A). Expression of specific genes during the differentiation of osteoclasts. CTSK, TRAP, RANKL and NFATc1 mRNA levels are suppressed by TEC (B). Inhibitory effects of TEC on NFATc1, CTSK and c‐Fos are shown in representative images (C). Quantification of band intensity ratio of NFATc1, CTSK and c‐Fos relative to β‐actin (D). Western blotting was used to analyse the primary antibodies against NF‐κB p65/p‐p65/IKB‐α. TEC inhibition of the NF‐κB pathway and promotion of IKB‐α downstream are shown (E). Quantitative analysis of p‐p65 was normalised to total p65. NF‐κB p65/p‐p65/IKB‐α phosphorylated levels were significantly suppressed by TEC from 20 min to 60 min. All bar graphs are presented as mean ± SD; *n* = 6. *p < 0:05, **P < 0.001, relative to the untreated group. #*p* < 0.05, ## *p* < 0.001, relative to RANKL treatment group.

We investigated the molecular mechanisms by which TEC modulates the process of osteoclast differentiation. We identified key genes that are specifically expressed during osteoclast formation. Following 3 or 5 days of TEC treatment and RANKL induction, the mRNA expression levels of osteoclast differentiation genes such as CTSK, RANK, TRAP and NFATc1 exhibited dose‐dependent inhibition (Figure 4A). Additionally, the protein levels of NFATC1, CTSK and c‐Fos were found to be significantly reduced (Figure 4B,C). To further explore the underlying mechanism, we examined the pathways associated with RANKL‐induced osteoclastogenesis (Figure 4D,E). The results revealed a significant decrease in the ratio of phosphorylated p65 to total p65 after 20 and 60 min of TEC treatment. The decrease in IĸB‐α after TEC treatment was similar to the decrease in NF‐ĸB. These results indicate that TEC inhibits signalling of NF‐ĸB and its downstream targets and inhibits IĸB‐α activity.

### 3.5 TEC Protects Against Bone Loss Caused by Glucocorticoids in C57BL/6J Mice

**FIGURE 5:**
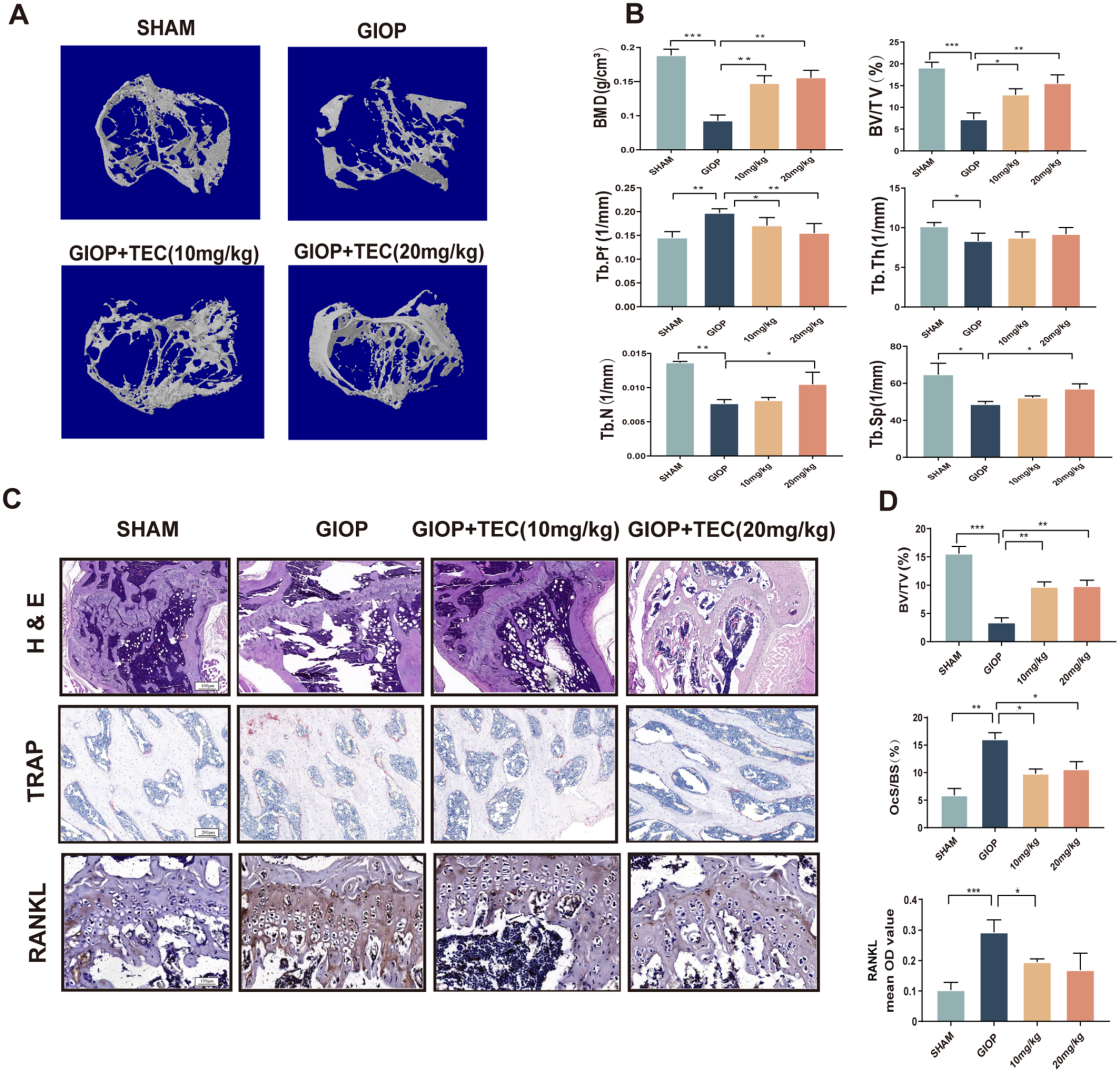
TEC reduces GIOP‐induced bone mass loss in vivo. (A) Tibial transverse views of the knee joint and Micro‐CT images of sagittal in different groups of mice. (B) Trabecular bone parameter analysis of BMD, BV/TV, Tb.N, Tb.Pf, Tb.Th and Tb.Sp. (C) H&E staining showed that the thickness bone trabecula surface is more maintained in TEC‐administration groups than GIOP group. Compared to TEC‐administration and Sham group in IHC staining, the sections in OVX group shows high expressions trend of RANKL, and the result also shows a dose‐dependent manner. The activity of osteoclast was assessed by TRAP staining. (D) The quantitative analyses of HE and TRAP staining, including BV/TV (*n* = 6) and OcS/BS (*n* = 6). All bar charts are presented as the mean ± SD. *p < 0.05, **p < 0.001 compared with the GIOP group.

Through the CT scan of the bilateral knee joint, we verified that the trabecular BMD, Tb.Th and Tb.N of the GIOP group reduced largely compared to the Sham group (Figure 5B), which indicated that the model establishment was successful [22]. The ratio of bone tissue volume to tissue volume (BV/TV), which can directly reflect the change in bone mass, is also an indirect index of bone mass; our results revealed the same tendency as BMD. Administration of TEC remarkably protected the animals from GIOP‐associated bone loss and trabecular deterioration both in the low‐ and high‐dose groups. The BMD, BV/TV, the number, thickness and separation of trabeculae (Tb.N, Tb.Th and Tb.sp) recovered after the administration of TEC (Figure 5B). It is worth noting that the trabecular pattern factor increased in the TEC groups, indicating that the trabecular bone changed from plate to rod. H&E staining revealed that bone volume and surface in GIOP mice were both well‐preserved after TEC treatment compared to the control group. The results of IHC staining showed that TEC had a positive effect on the expression of RANKL in the trabecula of tibial tissues, while the osteoclast surface area decreased in the TECdministration groups when compared to the GIOP group (Figure 5C). Collectively, TEC retarded the bone resorption, leading to the protective effect against GIOP‐associated bone loss.

### 3.6 TEC Inhibits Osteoclast Differentiation by Regulating Th17/Treg Balance

**FIGURE 6:**
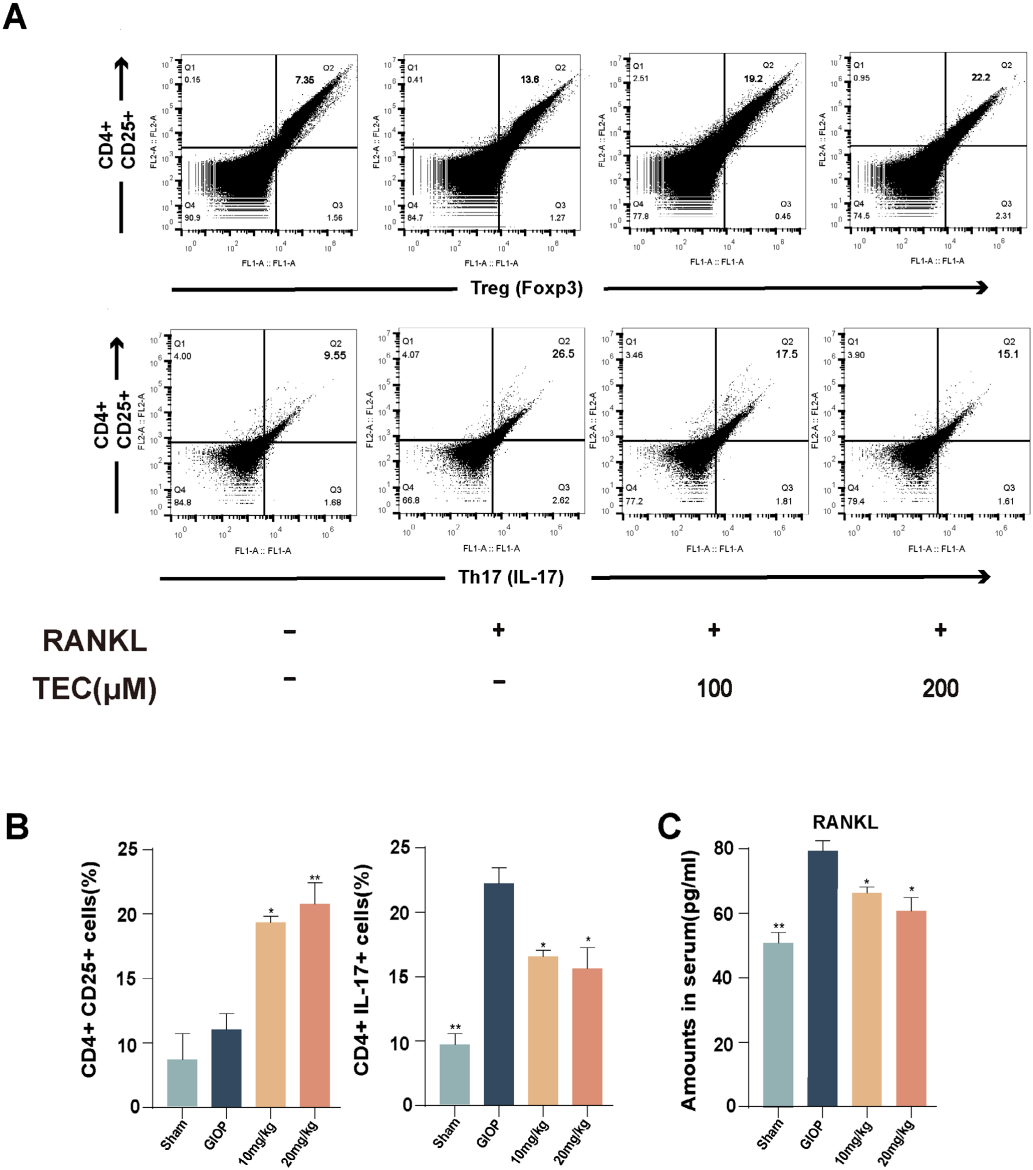
The effect of TEC on the differentiation of mouse spleen Th cells into Treg or Th17 cells. (A) Treg cells were stained for intracellular Foxp3 and surface CD4+ and CD25; Th17 cells were stained for intracellular IL‐17 and surface CD4 +, then analysed by flow cytometry. (B) The proportion of Treg and Th17 cells in the spleen of each group of mice. (C) The level of RANKL in mice serum was examined by ELISA kit. All values are expressed as mean ± SEM. *p < 0.05, **p < 0.001 versus model.

Bone resorption and RANKL overexpression during GIOP are closely associated with the local Th17/Treg imbalance. Thus, to assess whether the inhibitory effect of TEC on bone loss and RANKL‐mediated osteoclast activation was associated with the modulation of the Th17/Treg imbalance, the percentage of Th17 and Treg cells in splenocytes was analysed by flow cytometry assay (Figure 6). The result showed that the percentage of Th17 cells in GIOP mice was higher than in normal mice, while there was a reduction of Th17 cells in TEC‐injected mice compared with the percentage in GIOP mice (Figure 6A,B). On the contrary, the result indicated a significant reduction of Treg cells in GIOP mice as compared to normal mice, whereas the percentage of Treg cells in TEC‐injected mice was higher than in GIOP mice (Figure 6A,B). Concomitantly, ELISA results indicated that the plasma levels of RANKL in the GIOP group were notably augmented. Following treatment with the TEC injection, a notable diminution in RANKL levels was evident in the treated mice when juxtaposed against the corresponding values observed in the GIOP mice. Those data support that GIOP‐induced osteoporosis can cause a reduction of Treg cells and an increase in Th17 cells in the mice. With the concentration of TEC treatment, the Th17/Treg balance had been recovered, and this might be due to attenuating the GIOP‐induced osteoporosis.

## 4 Discussion

**FIGURE 7:**
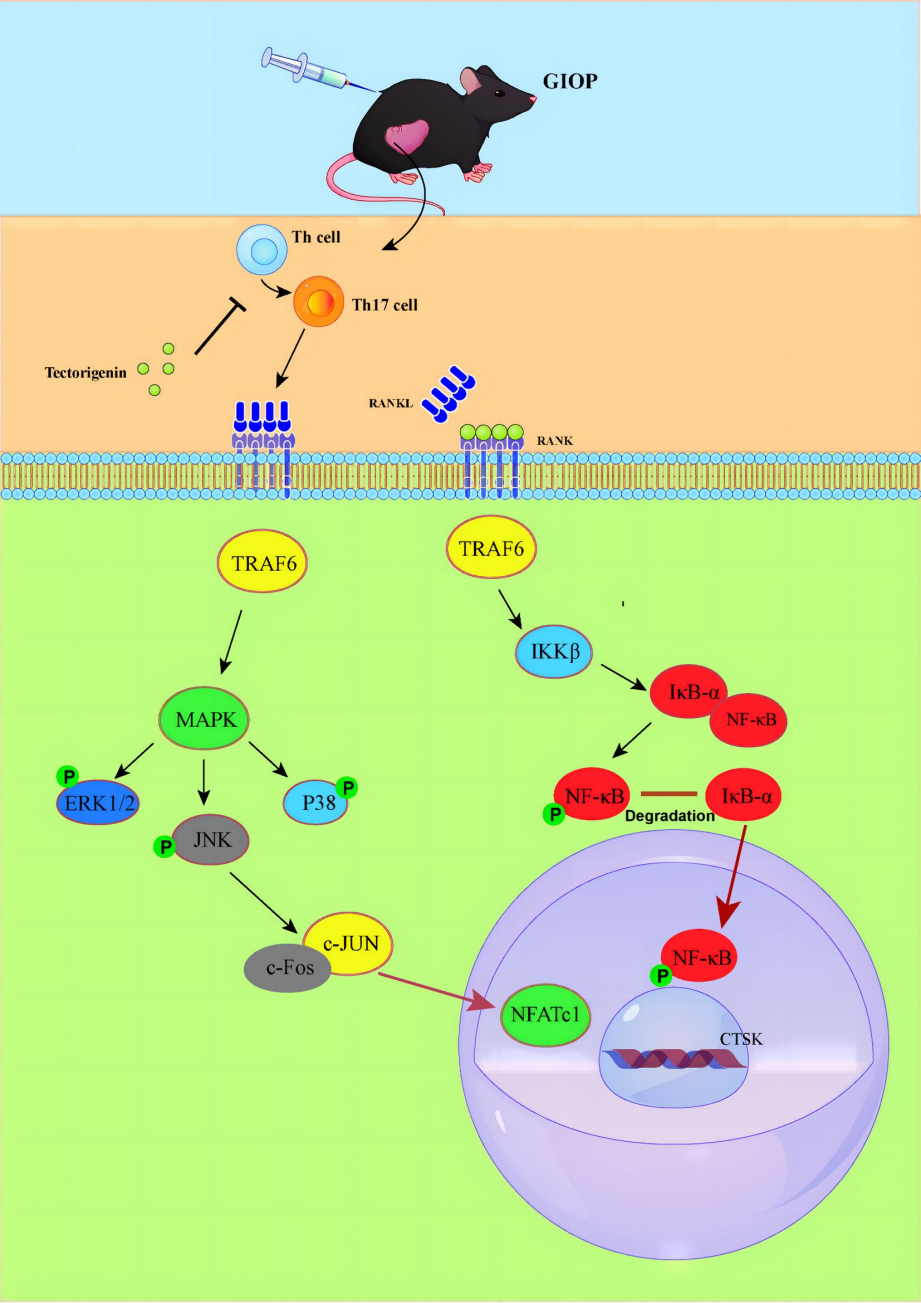
The mechanism of TEC inhibits GIOP mice osteoclast formation. Schematic representation of the molecular regulation of osteoclastogenesis induced by TEC and RANKL. TEC leads to attenuation of nuclear translocation and autoamplification of NFATc1 through inhibition of RANKL and RANK binding, MAPK and NF‐κB pathways and blockade of calcium oscillations. TEC attenuates nuclear translocation and auto‐amplification of NFATc1 by inhibiting RANKL and RANK binding, MAPK and NF‐κB pathways and blocking calcium oscillations. TRAF6, TNF receptor‐associated factor 6; MAPK, mitogen‐activated protein kinase; JNK, c‐Jun N‐terminal kinase; Erk, extracellular signal‐regulated kinase; NFATc1, nuclear factor 1 of activated *T* cells.

Excessive formation of osteoclasts and bone loss are the main causes of osteoporosis incidence [23]. Osteoblast‐mediated bone formation and osteoclast‐mediated bone resorption contribute to bone turnover and remodelling. Therefore, inhibiting osteoclast activity is an effective method to treat osteoporosis. In this study, we investigated the effect of TEC on GIOP in vivo for the first time, as well as its effects on the viability of osteoclasts. We found that the natural compound TEC can interact with RANKL and alleviate glucocorticoid‐induced bone resorption by suppressing osteoclast activity through the NF‐kB signalling pathway and Th17 cell differentiation (Figure 7).

To reveal the interactions between TEC and RANKL, molecular docking simulation was conducted. Our results indicated that TEC had a strong interaction with human RANKL/RANK complex, suggesting a close combination of RANKL and TEC. TEC may therefore be beneficial in preventing the formation of RANKL–RANK complexes. Additionally, RNA sequencing was performed, and differential expression genes and their bioinformatics analysis showed that it has an important impact on the osteoclast differentiation pathway. This further suggests that TEC may suppress osteoclast formation by binding RANKL. Multinucleate osteoclasts are characterised by high expression of the TRAP enzyme [24]. To evaluate the biological function of TEC, the number of osteoclasts induced by TEC was measured. In the TEC‐treated group, the number of osteoclasts decreased significantly in a dose‐dependent manner.

The NF‐kB pathway is one of the primary signalling pathways required for osteoclast differentiation induced by RANKL [25]. NF‐κB knockout animals exhibited problems with osteoclast development as well as severe osteoporosis [26]. In a relaxed state, NF‐κB is found in the cytoplasm as a non‐active form that is linked to the inhibitory protein IKB‐α. Additionally, application of glucocorticoid induces an inflammatory response and activates NF‐κB, a critical regulatory family involved in resistance, inflammation, cell survival and carcinogenesis. The activation of NF‐κB will aggravate inflammation. In this study, we discovered that GIOP raised inflammatory biomarkers such as NF‐κB p65/IKB‐α levels while TEC significantly reduced these inflammatory markers [27]. TEC has been previously demonstrated to suppress the production of pro‐inflammatory cytokines in RAW264.7 macrophages [28]. Based on the above results, TEC reduced RANKL‐induced osteoclast development through decreasing the NF‐κB signalling pathway.

As Arron and Choi proposed the concept of osteoimmunology in 2000, this cross‐disciplinary field has attracted great interest and attention [29, 30, 31]. Treg/Th17 cells play an important role in regulating bone homeostasis. Regulatory *T* cells (Treg cells) inhibit osteoclastogenesis and are known protectors of bone health. However, Th17 cells induce osteoclastogenesis and are involved in enhanced bone loss in osteoporosis. Also, Treg cells inhibit the differentiation of Th17 cells and vice versa [32]. In line with these findings, we report for the first time that TEC can regulate bone health via modulation of Treg/Th17 cell balance. Compared with the PC group, the TEC‐intervened group showed a significant increase in the population of Treg cells along with a simultaneous decrease in Th17 cell population. Taken together, these results clearly demonstrate that TEC can protect bone health by enhancing anti‐osteoclastogenic Treg cells and inhibiting osteoclastogenic Th17 cells. Previous studies show that the NF‐κB signalling pathway will influence the Treg/Th17 cell balance [33, 34]. Chen et al. demonstrated that miRNA‐148a‐containing GMSC‐derived EVs modulate Treg/Th17 balance via the IKKB/NF‐κB pathway and treat a rheumatoid arthritis [35].

Considering the effects of TEC in vitro, we further studied its effects in conditions of excessive RANKL generation in vivo. Previous studies have shown that glucocorticoids can up‐regulate the expression of RANKL and M‐CSF by directly acting on the osteoblasts, which is essential for osteoclastogenesis [7]. GIOP mammal models share some features with GIOP patients who undergo long‐term glucocorticoid therapy and experience significant bone loss due to excess glucocorticoid exposure, resulting primarily from trabecular bone loss [36, 37]. Trabecular bone micro‐architecture is an effective predictor for glucocorticoid‐induced bone loss and bone quality deterioration. Bone microCT parameters confirmed that TEC can ameliorate trabecular micro‐architecture destruction and prevent bone mass decrease in GIOP mice, suggesting that TEC might be a good candidate for the prevention and treatment of GIOP.

However, our study acknowledges limitations regarding TEC's long‐term safety and potential off‐target effects, which require further investigation. While TEC shows promise in treating osteoporosis, its mechanisms may differ from those of established drugs like bisphosphonates and Denosumab, necessitating comparative studies. Future research should focus on evaluating TEC's long‐term safety and clarifying its therapeutic mechanisms to determine its role in clinical practice.

## 5 Conclusion

In all, we described that TEC can affect osteoporosis via NF‐κB pathway positively. Meanwhile, TEC can protect against bone loss by decreasing osteoclastogenesis through NF‐κB downregulation and modulating Treg‐Th17 cell balance. This conclusion can provide a novel candidate drug for GIOP therapy with few adverse side effects and reveal a new perspective idea for TEC anti‐osteoporosis mechanism study.

## Author Contributions

**Peng Peng:** formal analysis (equal), investigation (equal), methodology (equal), visualization (equal), writing – original draft (equal). **Puiian Wong:** data curation (equal), formal analysis (equal), methodology (equal). **Zheng Lv:** data curation (equal), software (equal). **Jiaqing Tian:** software (equal), writing – review and editing (equal). **Wei He:** funding acquisition (equal), supervision (equal), writing – review and editing (equal). **Qiushi Wei:** funding acquisition (equal), supervision (equal), writing – review and editing (equal). **Hui Mo:** funding acquisition (equal), project administration (equal). **Mincong He:** funding acquisition (equal), supervision (equal), writing – review and editing (equal).

## Conflicts of Interest

The authors declare no conflicts of interest.

## Data Availability

Data will be made available on request.
